# Exploring Dog-Assisted Interventions in Higher Education: Students’ Attitudes and Perceived Effects on Well-Being

**DOI:** 10.3390/ijerph18094492

**Published:** 2021-04-23

**Authors:** Cathrin Rothkopf, Silke Schworm

**Affiliations:** Department of Educational Science, University of Regensburg, 93053 Regensburg, Germany; silke.schworm@ur.de

**Keywords:** human–animal interaction, animal-assisted intervention, higher education, at-risk students, attitude, interest, well-being, blood pressure

## Abstract

Both, in the transition to university and during it, students experience a multitude of different changes. Thus, it is no surprise that many students in higher education report suffering from mental health problems. To address their concerns, animal-assisted interventions (AAIs) have gained more and more attention over the past few years. Nonetheless, AAIs have neither yet been used nor researched at German universities. Two studies were carried out to address this issue. In Study I, 709 university students answered a questionnaire evaluating their attitude towards dogs, AAIs and interest in its use at their home university. In Study II, 27 students participated in a dog-assisted intervention (DAI) in which they were allowed to interact with a qualified dog for 15 min. To gain information about their well-being, blood pressure was measured and the Basler Befindlichkeitsskala had to be answered before and after the intervention. Results showed a positive attitude among German students toward dogs, AAIs, and the use of DAIs at their home university. Although an effect on physical well-being could not be found, results showed that a 15-min DAI can improve students’ psychological well-being. Thus, higher education administrators should consider using DAIs as a way to improve student well-being.

## 1. Introduction

Going to college or university can be challenging for many students worldwide. Not only do a lot of things change when entering university, but also new difficulties keep appearing. As a consequence, the occurrence of mental health issues in students in higher education is on the rise [1,2]. Over the last years, university students have reported experiencing, amongst others, increasingly high levels of academic stress, depressive symptomology, anxiety, and suicidal ideation [3,4] due to academic pressure, external and internal expectations, time and financial management, geographic locations, new social environments, and family circumstances [5,6,7,8]. According to Bewick et al. [9] students’ stress levels increase upon entry to university and does not decrease throughout their time there. High stress levels, in turn, have a negative impact on students’ learning capacity, academic performance, education and employment attainment, sleep quality and quantity, substance use, as well as on psychological and physical well-being [10].

A study by the German Centre for Higher Education Research and Science Studies (DZHW), the Freie Universität Berlin and the health insurance fund Techniker Krankenkasse (TK) revealed that several mental health issues can also be seen in students in Germany. Not only do they rate their state of health far worse than a comparison group of about the same age did [11], but their stress experience is also more distinctive compared to the average stress experience of employees [12] and they showed to have a higher psychological distress than the general population [13]. About a quarter of students (25.3%) experience high levels of stress, 24.4% were affected by exhaustion, and the prevalence for depressive syndrome and generalized anxiety disorder among the surveyed students was about one sixth, with no significant differences due to course of study, but a significantly higher proportion of highly-stressed female students. Not only psychological complaints appeared—burdensome and stressful living conditions of students in higher education can also result in various physical complaints (e.g., headache, limb, or back pain) due to somatization processes. Of all students, 73.8% suffer at least a few times per month, and half of the students (49.6%) once a week or more often from at least one somatic complaint [11]. To sum up, German students in higher education rate their state of health worse than their non-studying peers and have more frequent physical and psychological complaints–especially female students.

Rising numbers of students’ suffering from mental health problems result in an increased demand for counseling services [14]. The annual survey 2018–2019 of the Association for University and College Counseling Center Directors showed a mean student-to-counseling-staff ratio at colleges and universities of 1318:1 [15]. According to the German National Association for Student Affairs, 108.800 counseling contacts were recorded by the psychological counseling centers of the student services organizations in 2017 in Germany, which is an increase of 60% compared to 2006 [16]. However, the vast majority of students who are struggling with mental health issues do not receive any treatment [17] and are still refraining from seeking mental help from traditional college counseling services due to barriers such as lack of time, lack of confidentiality, stigma associated with using mental health services, lack of financial resources, fear of documentation on academic records, and fear of unwanted intervention [18,19]. Thus, alternative intervention and prevention programs need to be implemented to address students’ concerns and to help them manage this stressful life situation more effectively.

One approach to improve the well-being of students in higher education, which has seen a surge in implementation, is the use of animal-assisted interventions (AAIs). Due to its rising popularity, it has become a prevalent topic of discussion and area of research in recent years. Based on different meta-analytic reviews that have shown AAI to be effective in a variety of treatment settings [20,21,22,23], the use of AAIs has gained international recognition with programs expanding into several countries [24]. Its use on campuses has gained popularity since 2005 [25]. The majority of these—predominantly dog-assisted—university programs are free for students and universities as most of the dog handlers are volunteers. Prevalent formats of AAI programs in higher education involve either a group of about 4–5 students interacting with one animal, most common a dog, in the presence of its handler during a single drop-in session for about 10–20 min, or animals and their handlers have certain hours and places (often in counseling centers, libraries, pet-friendly dorms, or outdoors) where they are available to interact with the students. Moreover, longitudinal programs lasting several weeks, which include communication between a certain group of students with assigned dog teams and their dog handlers, are also possible. These AAI formats allow a far greater number of students to participate over a shorter period of time than other types of format that have been shown to have positive effects on the mental health and well-being of students, but which require a longer period of time and are more resource-intensive.

Although providing this kind of intervention to college and university students is a fairly new undertaking [26], there is promising evidence that AAIs can, amongst others, result in higher ratings of momentary positive emotions, a reduction in stress-related negative emotions [27,28], psychological and physical markers of stress [29,30,31,32], anxiety [33,34,35,36], and homesickness while increasing their satisfaction with life, connectedness to campus [37,38], behavioral aspects of academic success [39], well-being and improving their social skills as well as their mood [29,33,40]. Furthermore, Daltry and Mehr [41] found that having animals on campus may promote the services offered by student counseling centers on campus, as a lot of students were unaware of them before implementing the AAI. Thus, offering AAIs may be an effective way to get students in higher education to seek therapy in the first place. Nonetheless, research results are ambiguous and not completely consistent. While some studies, for example, report a reduction in physiological stress [34,42,43], others cannot confirm this effect [44,45]. However, as there is a clear tendency to confirm the positive effects of AAIs in higher education, even well-known universities, like Harvard Medical School or Yale Law School implemented AAIs on their campuses to benefit from them. By reducing, amongst others, symptoms of stress, anxiety and homesickness, these interventions may improve students’ mood, well-being, and academic success. Nevertheless, AAIs have not yet been introduced at German universities, nor have their effects on German students been examined.

Furthermore, little is known about students’ interest in and attitude towards AAIs. Some studies surveyed occupational therapists’, mental health workers’, nursing home staff’s, and inpatients’ attitudes towards AAI [46,47,48,49]. In general, the results showed a positive attitude towards AAI and its potential to have a positive impact on patients. In addition, staff members reported that they consider AAI an enrichment of their work and that it does not significantly increase the workload. Another study by Foreman et al. [50], evaluated employees’ attitudes about the impact of visitation dogs on a college campus. The results of their survey support a predominantly positive attitude, as staff felt that the dogs posed minimal risk and the vast majority of staff believed that they could reduce stress and provide comfort to students on campus. Students’ attitude towards the dog-assisted intervention (DAI) was not evaluated. However, a study by Pendry et al. [30] showed that students associate the combination of evidence-based academic stress management content and AAI with a higher degree of enjoyment, perceived benefit and likelihood of recommendation compared to content presentation or AAI alone. Yet, studies examining attitudes toward DAI in particular exist only in the context of Spanish and Romanian students [40,51,52], reporting a positive attitude and high interest in DAI. In addition, previous studies showed that attitude towards animals, AAIs, and interest in its use may be affected by social and demographical variables (such as gender, age, and religion) and also by psychological factors like personality traits [53,54,55,56,57,58,59]. A study investigating attitudes of German children and adolescents towards animals showed that gender and age are important factors influencing their attitudes. Girls showed a more positive attitude compared to boys, with positive attitude towards animals decreasing with age [60]. As there is a possibility that people who have a positive attitude towards animals may be motivated to believe that animals are beneficial for human health, it may influence interest in AAIs [61]. Crossman and Kazdin [62], for example, found that “individuals with positive attitudes toward companion animals perceived AAIs as more credible, acceptable, and positive, relative to individuals with more negative attitudes toward companion animals” (p. 1). However, if this also applies to students in higher education has not yet been investigated.

The aim of this work is to contribute to the current discussion about the implementation and the possible benefits of AAIs at colleges and universities. The two studies carried out in the context of this work complement the existing research on AAIs in higher education. While 62% of the surveyed universities in the Unites States have already implemented animal-assisted programs to receive the benefits deriving from them [26], this kind of intervention and prevention program has not been used in German universities up to now and little is known about German students’ interest in and attitude towards AAIs as well as its effect on their well-being. By evaluating German students’ interest in DAIs (Study I) and DAI’s effect on their current well-being (Study II), insights can be gained into target group acceptance, possible predictors and the effectiveness of DAIs in terms of improvement of psychological and physical well-being of German students. For this purpose, a questionnaire study, as well as an intervention study were conducted.

## 2. Study I: Materials and Methods

Study I was a questionnaire study. The main goal was to examine German students’ interest in and attitude towards DAIs. Additionally, based on the theoretical contexts presented, possible predictors were investigated. Thus, following research questions arise for the empirical analysis:

1. What attitude do German students have towards dogs, AAIs and the use of DAIs at their home university?
2. What are predictors for students’ attitude towards and interest in the use of DAIs at their home university?

Based on the presented theoretical background, our hypotheses were that German students have a positive attitude towards dogs, AAIs and the use of DAIs at their home university and that correlations between them exist. Furthermore, we expected that gender, field of study, age, attitude towards AAIs, and attitude towards dogs are predictors for students’ attitude towards and interest in the use of DAIs at their home university.

### 2.1. Design

For Study I, a quantitative questionnaire study in a cross-sectional design was chosen. The questionnaire consists of three translated and (if required) modified scales.

### 2.2. Participants

**Figure 1:**
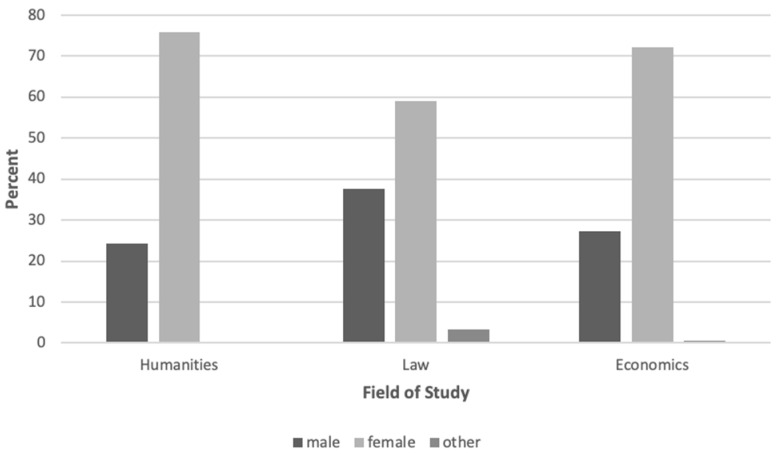
Participants’ gender distribution within the sampled faculties.

The target group of this study consists of students at a German university of three different fields of study. A total of 709 students participated in this survey. Of those 709 students, 200 identified themselves as male (28.2 percent), 496 as female (70 percent), 4 as other (0.6 percent), and 9 did not provide information on gender (1.3 percent). They were studying a range of subjects within the field of humanities (72.2 percent), law (17.2 percent), or economics (3.2 percent). The gender distribution of the sample within these faculties is shown in Figure 1.

Participants’ proportion of women was 75.8 percent in humanities, 59 percent in law, and 60.9 percent in economics. Reference proportion of female students in the evaluated year for sampled faculties was 76.59 percent in humanities, 60.3 percent in law, and 42.67 percent in economics [63]. Consequently, the sample provides a realistic representation of the gender distribution at this university. Their ages ranged from 16 to 40 years, with a mean age of *M* = 20.82 (*SD* = 2.69). At the time of data collection, the participants were on average in their third semester (*M*= 2.76, *SD* = 1.57). The students were recruited through visits in various courses and through social media websites. They did not receive any form of reward for participating in the study.

### 2.3. Instruments

The questionnaire consisted of a demographic survey and three scales addressing students’ attitudes towards dogs, AAT and the use of dogs on their campus. When selecting the measurement instruments, attention was paid to the fulfillment of the three essential quality criteria of reliability, objectivity and validity. Thus, the questionnaire consists of the Coleman Dog Attitude Scale (CDAS) by Coleman et al. [64], the Attitude Towards Animal-Assisted Therapy Scale (AATS) by Hightower [65], and the Cuestionario de Actitudes ante las Intervenciones Asistidas por Perros (CAINTAP) by López-Cepero et al. [52]. Due to its use at a German university, all questions had to be translated and some had to be modified for this context. In total, the questionnaire contained 70 items. All instruments included are described in the following.

#### 2.3.1. Demographic Survey

At the beginning of the questionnaire, participants were asked to provide demographic information on age, gender, field of study, and semester.

#### 2.3.2. Coleman Dog Attitude Scale (C-DAS)

The Coleman Dog Attitude Scale (CDAS) is a 24-item self-rating measure using a 5-point Likert-scale to assess attitudes towards dogs. It has proven to be reliable and valid with a Cronbach’s α ranging from 0.98 to 0.99 [64]. For this study, the original scaling was adjusted to the second possible predictor (attitude towards AAI) in order to allow better comparison. Thus, students were asked to rate their attitude towards dogs on a scale from 1 (strongly disagree) to 7 (strongly agree). Cronbach’s α obtained in this study is shown in Table 1.

#### 2.3.3. Attitude towards Animal-Assisted Therapy Scale Modified (AATS-M)

Consisting of 19 items, the Attitude Towards Animal-Assisted Therapy Scale measures the attitude towards animal-assisted therapy. The questionnaire showed a high internal consistency with a Cronbach’s α = 0.85 [65]. For its use in this study, some statements had to be modified to assess attitude towards AAI in the context of higher education and one item was added (“If animal-assisted pedagogy is to be introduced at the university, the animals should be trained for it”), resulting in a total number of 20 items. Participants were asked to rate their agreement of each statement on a 7-point Likert scale (1 = strongly disagree; 7 = strongly agree). Due to the modification of the AATS-M, the selectivity of each item of AATS-M was calculated and a factor analysis was carried out. The self-generated item as well as the items “I am familiar with animal-assisted intervention” and “only qualified dog handlers should be allowed to offer animal-assisted education at the university” were excluded from future analyses due to poor selectivity (*r* < 0.30). The Kaiser–Meyer–Olkin measure of sampling adequacy was 0.921 and the Bartlett’s test of Sphericity was significant (*χ^2^* (136, *N* = 709) = 5458.85, *p* < 0.001), indicating that the variables are suitable for factor analysis. Thus, a principal component analysis (PCA) with Varimax rotation was performed. The number of factors was determined using the Kaiser criterion and the Screeplot. A two-factor solution was obtained, explaining 53.4% of the overall variance. Based on their items, the two factors were named “approvals” (consisting of 10 items) and “objections” (consisting of 7 items). The resulting Cronbach’s α can be seen in Table 1.

#### 2.3.4. Cuestionario de Actitudes ante las Intervenciones Asistidas por Perros (CAINTAP)

The Cuestionario de Actitudes ante las Intervenciones Asistidas por Perros (CAINTAP) is based on the Brisbane Attitudes Towards Animal Assisted Therapy (Moody et al., 2002) and assesses students’ attitude towards DAI [52]. It consists of 22 items which are rated on a Likert scale ranging from 1 (strongly disagree) to 5 (strongly agree). Prior exploratory analysis showed a two-scale solution, evaluating positive (12 items) and negative attitudes (10 items) toward DAIs. It was validated for Spanish students at different public universities in occidental Andalusia and showed a reliability of Cronbach’s α = 0.879 for positive attitude and Cronbach’s α = 0.884 for negative attitude toward DAIs [52]. In this study, these factors were named “benefits” and “fears”. Obtained Cronbach’s α are shown in Table 1.

### 2.4. Procedure

The questionnaire was implemented both as an online and a printed version via Evasys, a professional and secure web-based platform for the design and conduction of paper, online, and hybrid surveys. The printed copies were distributed to the students at the beginning of different seminars and lectures within the field of humanities, law and economics during winter semester 2018/19 and summer semester 2019. To do so, various lecturers were asked for permission by e-mail beforehand. After a short introduction (including study goals, protection of anonymity and the possibility to cancel or withdraw participation) the questionnaire was handed out to the students. Participation was voluntary and took about 10–15 min. After approximately 20 min questionnaires were collected again. The online questionnaire was accessed through a link shared via various social media (e.g., Facebook groups, student council mail) and via QR code printed on flyers that were posted on notice boards at the university. Again, the survey began with a short introduction including information on study goals, protection of anonymity, the possibility to cancel or withdraw participation, duration of the survey and a declaration of consent. Most of the items were declared as mandatory answers. Students were pointed to the responsible students for further questions (email). The total of 709 completed questionnaires consisted of 668 printed and 41 online copies which were transferred to the IBM SPSS STATISTICS 24 statistical analysis software for analysis.

### 2.5. Analysis

In the first step, the data were transferred, clearly named and measurement levels of the variables were defined. Two C-DAS items, seven AATS-M items and 11 CAINTAP items had to be recoded and were stored in new items to allow for better interpretation of the results. In addition, the various study programs were summarized according to their respective fields of study (humanities, law, economics). Mean values, frequency distributions and a crosstab of relevant items for sample description were calculated.

To empirically test hypothesis 1, descriptive statistics were calculated. Therefore, the mean value indices of the scales C-DAS, AATS-M, CAINTAP, and their respective factors were determined.

Hypothesis 2 was examined using correlation analysis of the mean value indices of the scales C-DAS, AATS-M, and CAINTAP.

Due to the presumed relation between gender, field of study, age, attitude towards AAIs and dogs as predictors for students’ attitude towards and interest in the use of DAIs at their home university (hypothesis 3), a multiple linear regression was chosen. Students’ attitude towards and interest in the use of DAIs at their home university was surveyed via the CAINTAP, which consists of the two factors “benefits” and “fears”. Thus, two regression analyses were carried out, once with “benefits” (factor 1 of the CAINTAP) and once with “fears” (factor 2 of the CAINTAP) being the dependent variable. Gender, age, field of study, the C-DAS, and the two factors “approvals” and “objections” of the AATS-M were used as independent variables. First of all, it was investigated whether the prerequisites for the regression analyses were met. The normal distribution of the standardized residuals as well as homoskedasticity of the residuals from each regression was verified using graphic diagnostic methods (histogram, normal distribution diagram, and scatter diagram). Both histograms showed an acceptable image, with the regression containing the dependent variable “benefits” being slightly disturbed by single residuals at the right and left edges. In the normal distribution diagrams of each regression, the plotted pairs of values lied approximately on the line of the origin with a gradient of 1, allowing to assume a standard normal distribution of the residuals. The scatterplots revealed a random and homogeneous scattering of the residuals around zero. Therefore, homoskedasticity can be assumed. Furthermore, a test for multicollinearity was performed. Since all variance inflation factors (VIF values) were smaller than 10, a multicollinearity problem is not assumed. Each regression was calculated using the enter method. In addition, the effect size *f* was calculated according to Cohen [66]. To analyze how the significant categorical predictor “field of studies” affects the dependent variable “fears”, an ANOVA was conducted. Additionally, a post-hoc test (Tukey) was used to determine the differences of the mean values more accurately.

## 3. Results

### 3.1. German Students’ Attitude towards Dogs, AAIs and the Use of DAIs at Their Home University

Descriptive Statistics showed that students have a positive attitude towards dogs (M = 4.74, SD = 1.66), AAIs (M = 4.73, SD = 0.98) and the use of DAIs at their home university (M = 3.25, SD = 0.69). Looking at the factors, students show more approval (M = 4.74, SD = 1.22) toward AAIs than objections (M = 3.28, SD = 0.92). Furthermore, they see more benefits in the use of DAI at their home university (M = 3.52, SD = 0.79) than fears (M = 3.07, SD = 0.76). Consequently, hypothesis 1 can be confirmed.

### 3.2. Correlations between German Students’ Attitude towards Dogs, AAIs and the Use of DAIs at Their Home University

Correlation analysis demonstrated that attitude towards dogs, AAIs and attitude towards the use of DAI at students’ home university correlate significantly (Table 2). Thus, hypothesis 2 can be confirmed.

### 3.3. Predictors for Students’ AttitudeTtowards and Interest in the Use of DAIs at Their Home University

The linear multiple regression model including the dependent variable “benefits” was statistically significant (*F*(6, 624) = 318.78, *p* < 0.001), showing that these results would probably not have been obtained by chance. *R²* for the overall model was 0.75, indicating a high goodness-of-fit according to Cohen (1988). Thus, 75.4 percent of the variance of the criterion variable “benefits” is explained by the predictors used, which, according to Cohen (1992), corresponds to a strong effect (*f^2^* = 1.73). The analysis indicates that “approvals” (β = 0.57, *t*(624) = 20.18, *p* < 0.001) is the most influential, significant predictor in this model, while the least influential, significant and at the same time negatively influencing predictor is the variable “objections” (β = −0.14, *t*(624) = −5.77, *p* < 0.001). Another significant predictor was “attitude towards dogs” (β = 0.29, *t*(624) = 11.18, *p* < 0.001). Gender (β = −0.002, *t*(624) = −0.11, *p* = 0.913), age (β = −0.03, *t(*624) = −1.31, *p* = 0.192) and field of study (β = −0.01, *t*(624) = −0.29, *p* = 0.776), however, were not shown to be statistically significant predictors of the variable “benefits”.

For the dependent variable “fears”, the model also turned out to be significant (*F*(6, 624) = 83.86, *p* < 0.001), explaining 44.6 percent of the variance with a strong effect (*f^2^* = 0.90). The greatest weight for prediction in this multiple regression equation is given to the variable “objections” (β = 0.34, *t*(624) = 9.50, *p* < 0.001). The predictor “attitude towards dogs” provides the second highest explanatory contribution within this regression equation (β = −0.33, *t*(624) = −8.53, *p* < 0.001). Approvals (β = −0.13, *t*(624) = −3.06, *p* < 0.01), field of study (β = −0.09, *t*(624) = −2.85, *p* < 0.01) and age (β = −0.06, *t*(624) = −2.05, *p* < 0.05) showed to be significant, negative influencing predictors, with age being the least influential one. However, gender (β = 0.01, *t*(624) = 0.30, *p* = 0.765) was not shown to be a statistically significant predictor for the variable “fears”.

**Figure 2:**
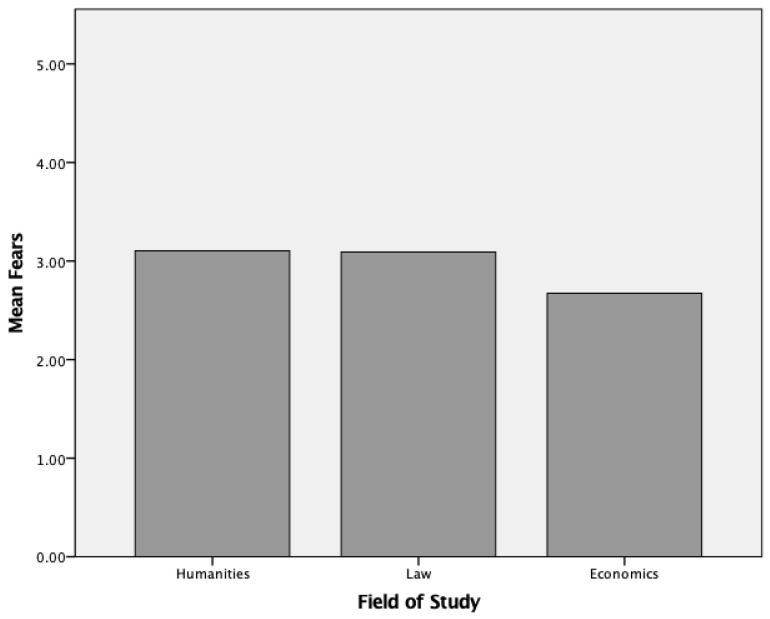
Differences within the factor “fears” due to field of study.

ANOVA including post-hoc test for the statistically significant predictor “field of study” revealed significant between-subject effects (*F*(2, 646) = 3.40, *p* < 0.05). Tukey post-hoc analysis demonstrated that there were significant differences (*p* < 0.05) between the field of study “economics” (*M* = 2.67, *SD* = 0.71) and the two fields of study “humanities” (*M* = 3.10, *SD* = 0.76) and “law” (*M* = 3.09, *SD* = 0.75) as shown in Figure 2. There were no significant differences between the field of humanities and the field of law (*p* = 0.984). In summary, hypothesis 3 can thus only be partially confirmed.

## 4. Study II: Materials and Methods

From the theoretical contexts presented, conclusions about the target group and the intervention design of this work can be drawn. AAIs have shown positive effects on physical and psychological well-being in a variety of settings. Study II was an intervention study and aimed at clarifying the extent to which this connection also exists in the context of university students in Germany. The general objective of Study II was to design, implement and evaluate the effects of a DAI on German students’ well-being. The design is based on prevalent formats of AAI programs in higher education abroad. A setting was chosen which on the one hand allowed individual observation, hence single setting, and on the other hand took animal welfare into account, hence 15 min of free interaction. Specifically, the research question is:

What effects do dog-assisted interventions have on German students’ psychological and physical well-being?

Based on the results of previous studies, we hypothesized that DAIs have a positive effect on students’ psychological and physical well-being.

As Study II is part of a more comprehensive study, only the information relevant to the research question is presented below.

### 4.1. Design

For this study, a one-group pretest-posttest design was used. It contained pre-post comparative measurements for the time before and after the DAI, but no control group.

### 4.2. Participants

From 36 registered students, 27 were randomly selected to participate in the study. 22 of them identified themselves as female, 5 of them as male. Their age ranged between 19 and 30 years (*M* = 23.00; *SD* = 3.15) and they came from different study programs (e.g., teacher-training course, media studies, sports science). However, most of the participants were from the teacher-training course (*n* = 11). All participants signed a written declaration of consent prior to participation in this study.

### 4.3. Instruments

To investigate effects on psychologic well-being, a questionnaire consisting, amongst others, of a demographic survey and the Basler Befindlichkeitsskala was compiled. Physical well-being was measured by blood pressure. All instruments are described in detail in the following.

#### 4.3.1. Demographic Survey

At the beginning of the questionnaire, participants were asked to provide demographic information, amongst others, on age, gender, and course of study.

#### 4.3.2. Basler Befindlichkeitsskala (BBS)

The psychological effect a DAI may have on students’ current well-being was measured using the Basler Befindlichkeitsskala (BBS), as it is well known for its sensitivity to short-term changes. It was developed by the Swiss Psychologist Viktor Hobi in 1985 to measure changes in subjective feelings of well-being (“Befindlichkeit”). The BBS is a standardized, German self-rating scale which is frequently used in settings of psychophysiological and psychopharmacological studies as well as in follow-up studies with healthy and mentally ill people. To assess current well-being, 16 pairs of adjectives arranged as a semantic differential are used. The bipolar word pairs (items) can be grouped into four factors, each consisting of four items [67]:

1. Vitality (well rested versus tired, fortified versus weakened, powerless versus energetic, healthy versus ill),
2. intrapsychic balance (unbalanced versus balanced, confident versus unconfident, calm versus nervous, anxious versus not anxious),
3. social extroversion (talkative versus discreet, reserved versus communicative, sociable versus shy, reclusive versus gregarious), and
4. vigilance (distracted versus attentive, vigilant versus absent-minded, concentrated versus inattentive, determined versus deflectable).

Participants were asked to indicate the extent to which statements described their current feelings on a 7-point scale (e.g., “Right now, I feel sociable … shy”). The completion of the questionnaire takes approximately 2–5 min. According to Hobi [68], each factor showed to have high Cronbach’s *α* ranging between *α* = 0.83 and *α* = 0.91, indicating good reliability and internal consistency. Cronbach’s *α* for the total inventory was reported to range between *α* = 0.92 and *α* = 0.95 for the healthy group and the sick group, respectively. In the context of the present study, the following Cronbach’s *α* were obtained before and after the DAI (Table 3).

#### 4.3.3. Blood Pressure

Systolic (SBP) and diastolic blood pressure (DBP) were measured using a Sanitas sphygmomanometer (blood pressure monitor). To have one single comparable value, and as it includes the effect of both, SBP and DBP, mean arterial pressure (MAP) was determined according to following equation:

MAP = DBP + 1/3(SBP − DBP).(1)

The MAP describes the average blood pressure in the aorta of an individual during a single cardiac cycle and is used to approximate the pressure gradient of the participants [68].

### 4.4. Procedure

Based on existing studies in the field of “AAI in higher education”, the approach and the implementation were planned, discussed, and organized. Dog–human teams suitable for data collection were contacted. These teams either were experts in DAIs or had already successfully completed a theory course on “Animal Assisted Pedagogy” and were tested for suitability by a trainer of the Mantrailing Working Group. To protect the animal and the participants, only teams that were tested or could prove their suitability for this purpose were used in this study. This includes the ability of the dog owners to read the facial expressions and body language of their dog, as this not only guarantees the protection of the dog, but also the participants’ [69]. In total, six of the selected dog–human teams agreed to participate in the study. They then were briefed about the study (aim of the study, procedure, required items) via e-mail. The owners were asked to bring their own dog toys and treats to ensure dog acceptance. Nine appointments for data collection were scheduled, with each allowing three persons to participate.

Once the dates were set, a Doodle survey was posted online with the basic information about this study, as well as the dates and time periods available. The study was announced in several seminars and via flyers on notice boards. After four weeks, the survey was closed and 27 of the 36 interested persons were selected by random drawing and notified via e-mail.

In this study, ethical standards for both humans and animals were considered as this is important when dealing with studies that involve humans and animals. To ensure the dog’s well-being, the room was checked for its adequacy (suitable size, surface, objects/furniture). The dog’s exposure to stressful situations was kept at a minimum. To guarantee this, the dog was observed both during and after the intervention by its owner, who was able to interpret the behavior and, in case of stress symptoms of the dog, had the possibility to stop the intervention immediately. It was also ensured that the dog had a safe haven (his blanket) and access to water. The time periods were also planned in a way which allowed a 15-min break between each intervention for the dog to recover, and, if necessary, to go for a walk. The total duration of 90 min per dog per day was therefore composed of a maximum of three interventions (15 min of intervention and 15 min of rest).

**Figure 3:**
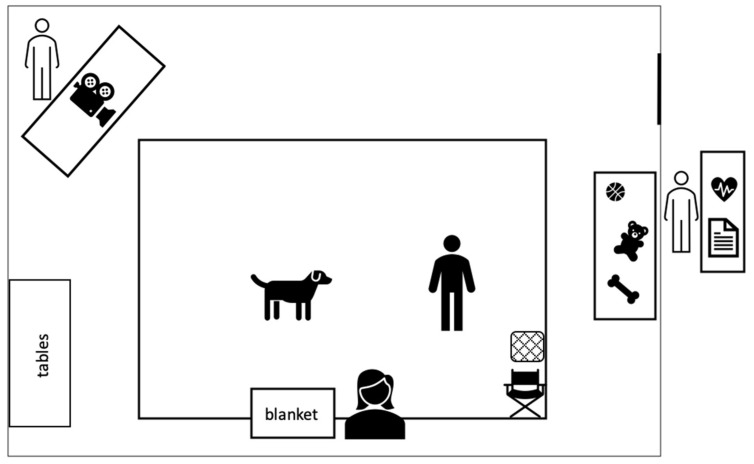
Design of the intervention room.

The room was prepared before the intervention began. In front of the room, one of the researchers was located with the questionnaires and the blood pressure monitor for data collection before and after the intervention. In the room, a mat, representing the intervention area, was laid out. Next to the door, a table was located with different toys and treats placed on it. By the wall, centered on the intervention area, a chair for the dog’s owner to sit on was placed. Next to him was the dog’s safe haven, the blanket. Diagonally from it, one of the researchers videotaped the intervention (these data will be analyzed in another study). In addition, the participants were offered a chair and a pillow. The design of the intervention room is shown in Figure 3.

The dog–human team was allowed to enter the room approximately 10 min before the start of the intervention to give the dog an opportunity to explore and acclimate to the new environment and to ensure that the participants did not yet have any contact with the dog prior to the intervention.

The participants received an information sheet (duration and procedure of the study, collected data, protection of anonymity, the possibility to cancel or withdraw participation) and a declaration of consent before the DAI. After that, blood pressure was measured and noted on the pre-questionnaire and the BBS (Pre) was handed out. Once the questionnaire was completed, a microphone was attached to the subject (these data will be analyzed in another study). To prepare the dog owner and the researcher, who started the video recording, the participant was requested to knock on the door before entering. While the owner introduced themselves and the dog and, in some circumstances, giving rules of behavior (e.g., do not lift the dog), the dog was still on a leash. Only after receiving permission, the leash was removed. During the intervention, the participant was allowed to interact freely with the dog (e.g., petting, playing, giving commands, giving treats). The handler was instructed to respond to questions, but to minimize conversation in order to avoid a potential disturbance or influence variable. Two minutes before the end of the intervention, the researcher present reminded them of the time remaining. After 15 min, the intervention was stopped and, outside the room, the pulse was measured and noted on the post-questionnaire. Then, BBS (post) was filled out by the participants. All 27 pre-post-questionnaires were transferred to the IBM SPSS STATISTICS 24 statistical analysis software for analysis.

### 4.5. Analysis

First, data was transferred, clearly named and measurement levels of the variables were defined. Eight items of the BBS had to be recoded and were stored in new items for both the pretest and the posttest, so that high values represent high well-being. Then, mean values and frequency distributions for sample description were calculated.

To empirically test hypothesis 1, a paired samples *t*-test was conducted. Hence, mean value indices of the BBS (overall well-being) and its respective factors [67] vitality (VT), intrapsychic balance (IB), social extroversion (SE), and vigilance (VG) had to be determined. To obtain an indication of the magnitude of the effect of the DAI, Cohen’s *d* effect sizes were calculated.

To analyze the effect of the DAI on students’ physical well-being (hypothesis 2), MAP was determined, and a paired sample t-test was conducted.

## 5. Results

### 5.1. Effects of Dog-Assisted Interventions on Students’ Psychological Well-Being

The results showed that participants had a higher overall well-being after the DAI (*M* = 5.73, *SD* = 1.12) than before (*M* = 4.87, *SD* = 0.68). A paired-samples *t*-test found this difference to be significant (*t*(26) = 4.94, *p* < 0.001, *d* = 0.95). Significant improvement (*p* < 0.05) was also found in all BBS factors (Table 4). Thus, hypothesis 1 can be confirmed.

### 5.2. Effects of Dog-Assisted Interventions on Students’ Physical Well-Being

Paired sample *t*-test showed that there was no significant difference in MAP-values before and after the DAI (*t*(27) = 1.520, *p* = 0.143). Consequently, hypothesis 2 has to be rejected.

## 6. Discussion

The popularity of AAIs has increased steadily in recent years as their ability to improve the health and well-being of certain groups has already been demonstrated in several studies. However, depending on the country, there are still large differences in acceptance and knowledge regarding AAIs [51]. Since the use of AAIs in German higher education has not yet been researched nor implemented, the present studies are a supplementation to existing research. For this purpose, existing questionnaires were modified and intervention designs of universities already using DAIs were taken as models and adapted to the objects of this work. In Study I, the attitudes of German students regarding the use of DAIs at their home university, as well as possible predictors, were investigated. Since the transition to university is characterized, among other things, by sharp declines in psychological well-being, which do not dissipate even in the later stages e.g., [70], the aim of Study II was to investigate the potential effects DAIs may have on the physical and psychological well-being of students in higher education.

Results revealed that students in Germany have a positive attitude towards dogs, AAIs and the use of DAIs at their home university. These results regarding their positive attitude towards its use at students’ home university are in line with the results gained for Spanish and Romanian students [40,51,52]. Moreover, approvals of AAIs and the benefits they see in the use of DAIs at their home university were rated higher than objections and fears. Positive correlations were found between students’ attitude towards dogs, AAIs and the use of DAIs at their home university. Possible predictors of students’ attitudes toward the use of DAIs at their home university were examined for the respective factor “benefits” and “fears”. Thereby, it was shown that approvals, attitude towards dogs and objections are predictors for the benefits they see while an effect of gender, age and field of study could not be determined. The more positive the attitude towards dogs and the higher the approvals of AAIs, the more benefits they saw in the implementation of a DAI at their home university. In contrast, the higher the objections, the lower the perceived benefits of its use. Fears, on the other hand, were predicted by objections, attitudes toward dogs, approvals, field of study and age. The higher the objections and the more negative the attitude towards dogs and the lower the approvals of AAIs, the higher the fears regarding the use of DAIs at their home university. Similar results were found in elderly citizens’ interest in participation in an AAA program [71]. Pet attitudes significantly predicted their willingness to participate. Another study by Crossman and Kazdin [62], who investigated the influence of attitude toward companion animals on perceptions of AAIs showed that individuals with positive attitudes toward companion animals perceived AAIs more positively than individuals with more negative attitudes toward companion animals.

A comparison of fields of study revealed that students in the field of economics fear the use of DAIs at their home university less than students in the field of study law and the field of humanities. A reason for this could be their personal epistemology that might prevail within the field of study. These beliefs are not only distinguished by discipline (e.g., mathematics, science) but also by judgment domain (e.g., personal taste, meaning) [72] and may influence attitudes, as students can refer to them when critically reflecting on DAIs to assess what to believe and which knowledge claims to support [73] and which are significant for dealing with scientific findings [74]. Epistemological beliefs can thus be viewed as a lens through which individuals interpret information, set standards, and decide on an appropriate course of evaluation and action [75]. Moreover, fears were shown to decrease with increasing age. Existing research confirms the influence of age on fears [76]. Again, there was no effect of gender, which is in line with the results concerning Spanish students’ attitude towards AAIs [52].

Furthermore, results indicate that 15-min DAI affects students’ psychological well-being positively. These results support previous findings on the benefits derived from DAI in terms of improved psychological well-being [31,33,38]. Improvements in vitality, intrapsychic balance, social extroversion as well as for vigilance could be found. One reason for the positive effect animals may have on psychological well-being is their function as social support. A positive feedback loop of oxytocin was found between dogs and humans [77,78], meaning that the release of oxytocin leads to actions that stimulate an even greater release of oxytocin. This hormone plays an important role in the development of human bonds and is linked to an increase of pro-social behavior [79]. Oxytocin effects can be triggered in response to single encounters with animals. However, stronger and long-lasting effects are associated with stable relationships with animals, such as pet ownership, due to repeated exposure to oxytocin. In addition, human-animal bonding may be crucial for oxytocin release and oxytocin-mediated effects. Thus, the effects are stronger when a dog is familiar [80]. Another aspect in this context is the relaxing influence dogs have on humans. A reduction in cortisol levels, a stress hormone, has been found repeatedly in dog–human interactions [81,82]. In this way, the DAI may have affected students’ well-being positively.

Nevertheless, an effect on students’ physical well-being did not occur in this study. The influence dogs have on students’ physical well-being cannot be unambiguously answered, as some studies have linked DAI in higher education to improvements in health factors such as lower blood pressure and cortisol levels [32,42,43,81], while other studies have failed to confirm such effects [29,36,44].

Overall, these results provide evidence that DAIs in higher education have a valuable impact on students’ subjective well-being, whether physically measurable or not. Living creatures provide a pleasant external focus for attention and have a calming and relaxing effect on humans [83]. Since many people do not find as many opportunities to interact with animals and nature, AAIs in higher education can be a way to connect to the human evolutionary history (biophilia) and, hence, may enhance students’ well-being.

Despite the best of intentions, the studies conducted in this work were not without limitations. In both studies, the majority of participants were female. This, however, can be attributed to the general gender distribution at the university. Furthermore, females have been found to be more susceptible to stress and anxiety [11,84], which in turn may explain their interest in the DAI. Nevertheless, it must be pointed out that these results are thus not generalizable for men. In addition, the fields of study were not distributed evenly in Study I. Due to the small number of participants from the field of economics it is not possible to generalize the results regarding the effect of field of study.

Although the sample in Study II is representative of students who are interested in DAI and whose participation in this type of intervention is likely, it may miss a significant portion of the target group. Thus, this study may have focused primarily on students who are willing to participate in DAIs and who may have some belief in the positive effects of such a program. Hence, an influence of social desirability cannot be entirely excluded either. Also, possible influences of the intervention design (two observers, video recording, microphone) must be taken into account, which resulted in an unnatural atmosphere. The feeling of “being observed” may have increased the tension and, thus, could have resulted in the non-appearance of an effect on physical well-being (blood pressure). In addition, the design lacked a control group. Consequently, it cannot be assumed that the observed changes are exclusively resulting from the intervention, since the variables cannot be compared with a group without or with a different type of intervention. Additionally, the sample size was small and the observed effect on psychological well-being is limited to a short period of time (before to after the DAI). Thus, conclusions about medium- or long-term effects cannot be drawn and results must be interpreted cautiously. The low reliability for the post-test of the factor “intrapsychic balance” needs to be mentioned, too. This could be due to the fact that the variance has become very small (ceiling effect). Hence, the remaining variance is not systematic enough. Furthermore, Cronbach’s alpha values for the factor “Vitality” are low which might be due to the low number of questions and participants.

Furthermore, it is evident that student attitudes and well-being are influenced by variables other than those examined in these studies, as described earlier in the theoretical background. Therefore, it is important to recognize that although these findings are promising, further research is needed to better understand the underlying mechanisms of action as well as the role of structured implementation approaches. More research is needed to determine the influence of field of study regarding attitudes toward DAI, what types of interactions with the animal are most effective, how long sessions should last, how often sessions should be offered, whether DAIs effect students’ physical well-being or not, how long the psychological benefits last after the intervention and if this may affect academic performance.

## 7. Conclusions

To the best of the researchers’ knowledge, the present studies contribute to filling a research gap regarding German students’ attitudes toward the use of DAI at their home university and DAI’s effect on their well-being. The results indicated a positive attitude among German students toward dogs, AAIs, and the use of DAIs at their home university. In addition, the results of Study II provided further support that allowing students to interact with dogs on campus has positive effects on students’ well-being. Although an effect on physical well-being could not be found, results showed that a 15-min interaction with a dog can improve students’ psychological well-being. University administrators should thus consider providing DAIs to students in higher education as a way to improve their well-being, as DAIs would be easily accessible, short in duration, and cost-effective. DAIs would offer a new approach unencumbered by the stigmas typically associated with counseling, and could, therefore, facilitate students’ access to (psychological) help. Nevertheless, students’ fears must be considered, and solutions need to be worked out. The development and research of animal-assisted interventions in Germany is still in its infancy. Therefore, standardized definitions and guidelines, as well as certified training in this field need to be established.

## Figures and Tables

**Table 1:** Cronbach’s α for all Scales and Factors used in Study I.

	Cronbach’s *α*
**C-DAS**	0.98
**AATS-M**	0.91
Approvals	0.93
Objections	0.71
**CAINTAP**	0.93
Benefits	0.93
Fears	0.87

**Table 2:** Correlations between the scales used.

Correlations				
		Attitude towards Dogs	Attitude towards AAI	Attitude towards and Interest in DAI at Home University
Attitude towards dogs	Person Correlation	1	0.65 ^1^	0.72 ^1^
	Sig. (2-tailed)		0.000	0.000
	N	705	694	693
Attitude towards AAI	Person Correlation	0.65 ^1^	1	0.81 ^1^
	Sig. (2-tailed)	0.000		0.000
	N	694	697	689
Attitude towards and interest in DAI at Home University	Person Correlation	0.72 ^1^	0.81 ^1^	1
	Sig. (2-tailed)	0.000	0.000	
	N	693	689	696

^1^*p* < 0.01.

**Table 3:** Cronbach’s α for BBS and its respective factors.

	Pre-Cronbach’s *α*	Post-Cronbach’s *α*
**Basler Befindlichkeitsskala (16 Items)**	0.85	0.84
Vitality (4 Items)	0.60	0.54
Intrapsychic balance (4 Items)	0.77	0.15
Social extroversion (4 Items)	0.85	0.90
Vigilance (4 Items)	0.77	0.81

**Table 4:** Paired samples *t*-Test results of the DAI.

	*M*	*SD*	*t (df)*	*d*
POST Overall Well-Being	5.73	1.12	4.94 (26) ^1^	0.95
PRE Overall Well-Being	4.87	0.68		
POST Vitality	5.52	0.69	6.48 (26) ^1^	1.25
PRE Vitality	4.69	0.84		
POST Intrapsychic Balance	6.74	3.12	2.47 (26) ^3^	0.48
PRE Intrapsychic Balance	5.39	0.93		
POST Social Extroversion	5.24	0.97	3.41 (26) ^2^	0.66
PRE Social Extroversion	4.64	0.99		
POST Vigilance	5.37	0.86	4.60 (26) ^1^	0.88
PRE Vigilance	4.77	0.97		

^1^*p* < 0.001, ^2^ *p* < 0.01, ^3^ *p* < 0.05.

## Data Availability

Data available on request due to restrictions e.g., privacy or ethical.
